# Retraction Note: NF-κB maintains the stemness of colon cancer cells by downregulating miR-195- 5p/497–5p and upregulating MCM2

**DOI:** 10.1186/s13046-023-02812-z

**Published:** 2023-09-01

**Authors:** Longgang Wang, Jinxiang Guo, Jin Zhou, Dongyang Wang, Xiuwen Kang, Lei Zhou

**Affiliations:** 1 Department of Gastrointestinal Surgery, Shandong Cancer Hospital and Institute, Shandong First Medical University, Shandong Academy of Medical Sciences, 250117 Jinan, China; 2 Department of Respiratory Medicine, Taian Municipal Hospital, 271000 Taian, China; 3 Department of Endocrinology, Affiliated Yantai Yuhuangding Hospital of QingdaoUniversity Medical, 264000 Yantai, China; 4 Department of Endoscopy, Shandong Cancer Hospital and Institute, Shandong First Medical University, Shandong Academy of Medical Sciences, 250117 Jinan, China; 5 Department of Intensive Care Unit, The First People’s Hospital of Lianyungang, 222000 Lianyungang, China; 6 Department of Oncological Surgery, Shandong Cancer Hospital and Institute, Shandong First Medical University, Shandong Academy of Medical Sciences, No. 440, Jiyan Road, Huaiyin District, 250117 Jinan, Shandong Province China

**Retraction Note: *J Exp Clin Cancer Res*** **39, 225 (2020)**

10.1186/s13046-020-01704-w

The authors have retracted this article because the Western blot data presented in a number of the figures are unreliable. Longgang Wang, Jinxiang Guo, Xiuwen Kang and Lei Zhou agree with this retraction. Dongyang Wang has not responded to correspondence from the Publisher about this retraction. Jin Zhou has stated that she was not involved in the preparation and handling of this manuscript, she was not informed about its publication, and she did not grant permission for the use of her name and affiliation in this article.

